# Editorial: Health benefit and promotion of 24-hour movement behaviors in children and adolescents

**DOI:** 10.3389/fped.2025.1637442

**Published:** 2025-06-16

**Authors:** Yi Sun, Bowen Song

**Affiliations:** College of Physical Education, Ludong University, Yantai, China

**Keywords:** children and adolescent, family, school, society, 24-h movement behaviors

**Editorial on the Research Topic**
Health benefit and promotion of 24-hour movement behaviors in children and adolescents

## Introduction

The healthy development of children and adolescents is a focal point of societal concern. As a core determinant, 24-h movement behaviors—comprising sleep, sedentary behavior, and physical activity—play a crucial role in the synergistic development of physical growth, mental health, and cognitive function (1). However, global health monitoring data indicate that most children and adolescents fail to meet the recommendations of international 24-h movement behavior guidelines (2). This is accompanied by rising obesity rates, earlier onset of metabolic diseases, and other serious issues (3), underscoring the urgency of deepening research and strengthening interventions.

This special issue focuses on the 24-h movement behaviors of children and adolescents, systematically analyzing key influencing factors at the family, school, and society.

Families, as the primary environment for child and adolescent development, significantly shapes behavioral patterns through its structural characteristics and lifestyle. Fan et al. found that children from nuclear families and only children are more likely to meet 24-h movement guidelines. Liu et al. further demonstrated that family physical activity environments (including factors such as availability of sports equipment and parental modeling of exercise) can indirectly enhance preschoolers’ gross motor skills by increasing their moderate-to-vigorous physical activity levels. Notably, the widespread use of electronic devices has led to a substantial increase in screen time among children and adolescents, displacing physical activity and prolonging sedentary behavior. Wang et al.’s study on children with disabilities revealed a significant positive correlation between physical activity and vitamin D levels, whereas screen time was negatively correlated with vitamin D levels, providing critical evidence for health interventions aimed at limiting screen exposure.

Schools, as key settings for children's daily activities, have seen their effectiveness in educational interventions grow alongside the advancement of health promotion concepts. Guo et al.’s pioneering systematic meta-analysis showed that school-led behavioral interventions effectively increased students' moderate-intensity physical activity levels but had limited success in reducing sedentary behavior. Current interventions predominantly focus on “incremental” activity promotion, while “replacement-based” sedentary behavior interventions (e.g., standing classrooms) remain underexplored, highlighting the need for innovative strategies.

Societies exhibit complex interactive mechanisms in influencing children's movement behaviors, with socioeconomic status (SES) and urban-rural disparities serving as core explanatory variables. Studies by Zhang et al. and Fan et al. both confirmed that adolescents from higher-SES families engage in higher levels of physical activity. Conversely, Oginni et al. found that rural children outperformed their urban counterparts in physical activity volume and gross motor skill development, whereas urban children exhibited longer sedentary time, illustrating the profound impact of environmental factors on behavioral patterns.

Although existing research has established a relatively systematic theoretical framework, significant room for improvement remains in methodology and practical application. Future studies could advance in four key dimensions:

1. Strengthening longitudinal research designs to track the evolving health effects of 24-h movement behaviors and elucidate the dynamic mechanisms of family, school, and socioeconomic factors;
2. Developing equity-focused intervention strategies by optimizing public sports resources, innovating community health programs, and enhancing school health promotion initiatives to bridge health behavior gaps caused by socioeconomic disparities;
3. Advancing cross-cultural comparative studies to explore how cultural values shape movement behaviors and develop culturally adaptive intervention programs;
4. Constructing a multidimensional collaborative intervention system through family-school-community linkages to create a comprehensive supportive environment for healthy behaviors.

In conclusion, research on 24-h movement behaviors in children and adolescents holds significant academic value and carries the practical mission of improving population health. By deepening mechanistic studies, innovating intervention strategies, and strengthening cross-cultural collaboration, we can build a more scientific and effective support system for the healthy development of children and adolescents.
